# Short message service (SMS)-based intervention targeting alcohol consumption among university students: study protocol of a randomized controlled trial

**DOI:** 10.1186/s13063-017-1898-3

**Published:** 2017-04-04

**Authors:** Kristin Thomas, Marcus Bendtsen, Catharina Linderoth, Nadine Karlsson, Preben Bendtsen, Ulrika Müssener

**Affiliations:** 1 Department of Medical and Health Sciences, Faculty of Medicine and Health, Linköping University, Linköping, Sweden; 2 Department of Computer and Information Science, Science and Engineering, Linköping University, Linköping, Sweden

**Keywords:** SMS intervention, Student population, Randomized controlled trial, Alcohol consumption

## Abstract

**Background:**

Despite significant health risks, heavy drinking of alcohol among university students is a widespread problem; excessive drinking is part of the social norm. A growing number of studies indicate that short message service (SMS)-based interventions are cost-effective, accessible, require limited effort by users, and can enable continuous, real-time, brief support in real-world settings. Although there is emerging evidence for the effect of SMS-based interventions in reducing alcohol consumption, more research is needed. This study aims to test the effectiveness of a newly developed SMS-based intervention targeting excessive alcohol consumption among university and college students in Sweden.

**Methods:**

The study is a two-arm randomized controlled trial with an intervention (SMS programme) and a control (treatment as usual) group. Outcome measures will be investigated at baseline and at 3-month follow up. The primary outcome is total weekly alcohol consumption. Secondary outcomes are frequency of heavy episodic drinking, highest estimated blood alcohol concentration and number of negative consequences due to excessive drinking.

**Discussion:**

This study contributes knowledge on the effect of automatized SMS support to reduce excessive drinking among students compared with existing support such as Student Health Centres.

**Trial registration:**

ISRCTN.com, ISRCTN95054707. Registered on 31 August 2016.

**Electronic supplementary material:**

The online version of this article (doi:10.1186/s13063-017-1898-3) contains supplementary material, which is available to authorized users.

## Background

A growing proportion of the global burden of disease is due to excessive drinking of alcohol. Alcohol-related deaths increased by 30%, or about 5 million, between 1990 and 2010 [1]. Despite significant health risks, heavy drinking of alcohol among university students is a widespread problem and excessive drinking is part of the social norm [2, 3]. About every second young adult in Sweden attends university, making the health and wellbeing of this group an important public health concern [4]. Furthermore, alcohol habits that are formed during young adulthood have been found to influence future drinking patterns [5]. Student health services can offer advice and support for students who wish to reduce or quit their drinking; however, only a small proportion of students seek alcohol-related support [6]. It is thus important to generate effective interventions that have the potential to reach a large proportion of this population.

A growing number of studies suggest that short message service (SMS)-based interventions are cost-effective in terms of reaching a large number of people. SMS interventions have been shown to be effective in supporting behavioural change, weight loss, smoking cessation and management of diabetes mellitus [7, 8]. Overall, SMS can have a number of advantages compared with face-to-face interventions, such as high accessibility whereby messages are likely to be read within minutes, the receiving and reading of messages that requires limited effort, and the enabling of continuous, real-time, brief support in real-world settings or in contexts when the individual is the most vulnerable to drinking [9–12]. SMS-based interventions have been designed based on current evidence-based interventions and have adopted techniques similar to face-to-face treatments, for example, tailored advice or goal-setting [9, 13].

Several reviews have been conducted on the effectiveness of SMS-based interventions in reducing alcohol consumption. A recent review by Berman and colleagues [14] on mobile interventions targeting risky drinking among students concluded that the evidence on this type of intervention is unclear. The review included interventions based on SMS, interactive voice response and smartphone application. In two of the four studies that included SMS-based interventions there were nominal differences between the intervention and comparison groups, and in one study only there were differences among women. However, these studies consisted of brief interventions with a limited number of messages; few included control groups without prompts.

Another review looking at text message interventions only identified fourteen studies of which three solely looked at alcohol use among adolescents and young adults. The authors concluded that interventions that use text messages have a small positive effect on reducing substance use [7]. A recent review by Fowler and colleagues [15] identified eight studies that described mobile-based interventions aiming to reduce alcohol use among adults. Most of these interventions entailed text messages or smartphone applications and the majority suggested positive effects on the reduction of alcohol consumption. The authors concluded that although these are promising findings, they are preliminary because the studies involved small sample sizes and short-term follow up, and varied intervention content, regimens and duration. Thus, although there is emerging support for the effect of SMS-based interventions in reducing alcohol consumption, more research is needed.

This article presents the study protocol of a randomized controlled trial (RCT) testing the effectiveness of a newly developed SMS-based intervention targeting excessive alcohol consumption among college and university students in Sweden. The research builds on previous work from the AMADEUS research programme [10, 16, 17] (Thomas et al., in press).

## Methods

### Design

**Fig. 1:**
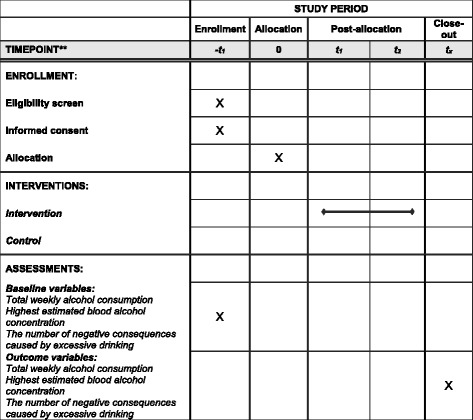
Standard protocol items: recommendation for interventional trials (SPIRIT). Schedule of enrollment, interventions and assessments. *SMS* short message service

The study is a two-arm RCT. Participants will be randomized to the intervention (group 1) or care as usual (group 2). Outcome measures will be investigated by questionnaires administered at baseline and after the intervention. The post-intervention follow up will be sent out simultaneously to all participants 3 months after the first study invitation. The control group will gain access to the intervention after the follow up. The study will be performed simultaneously at 14 universities in Sweden. Additional files 1 and 2 include a *Standard protocol items: recommendation for interventional trials* (SPIRIT) checklist for the schedule of enrollment, interventions, and assessments as presented in Fig. 1.

### Outcome measures

The primary outcome measure will be total weekly alcohol consumption. This is computed as the sum of alcohol consumption for each of the 7 days in a typical week. Secondary outcomes will be frequency of heavy episodic drinking (HED), highest estimated blood alcohol concentration (eBAC) on one drinking occasion and the number of negative consequences caused by excessive drinking. All measures are based on the past month.

### Objectives and hypotheses

The aim of the study is to test the direct effect of a newly developed SMS-based intervention targeting excessive alcohol consumption among university students in Sweden. The primary hypothesis is that participants in the intervention group will report significantly lower total weekly alcohol consumption at follow up compared with participants in the control group. Secondary hypotheses are that the intervention group will report a significantly lower frequency of HED, significantly lower eBAC and significantly fewer negative consequences caused by excessive drinking compared with the control group.

### Intervention group

The intervention will consist of a 6-week automated SMS-based programme with a total of 62 messages (Thomas et al., in press). The intervention was developed using formative methods including focus groups with students, an expert panel with students and professionals and behavioural change technique analysis [18]. The content of the SMS messages will include facts about the negative consequences of alcohol, tips on behavioural change strategies and activities such as saying no to alcohol. Additional file 1 includes all the SMS messages in the programme and the development of the programme has been described previously (Thomas et al., in press).

### Control group

The control group will be offered conventional care. At present, the typical practice at the Student Health Centre (SHC), besides person-to-person motivating advice, is to recommend a website to the students where they can estimate their alcohol consumption and receive feedback on their drinking levels and more information on the health consequences of drinking. Guidance such as this is currently distributed to students via email from the SHCs. Participants in the control group will be informed by SMS that they have been allocated to the control group, and will gain access to the SMS-based support once the main trial has ended. The SMS will also include tips on websites typically used by the SHCs. No additional prompts or reminders about the websites will be given during the study.

### Randomisation

Participants will be randomized to the intervention (group 1) or treatment as usual (group 2). Each participant will be allocated a number 1 or 2 with equal probabilities using the Java built-in random number generator (java.util.Random). Randomisation is thus fully computerized, does not use any strata or blocks, and is not possible to subvert, because this and all subsequent study processes are fully automated. The participants will know that they have been randomized to either an intervention or control group.

### Participants and procedure

All students at 14 universities and colleges in Sweden will be invited to take part. Inclusion criteria for students are drinking at least four standard drinks (women) or five standard drinks (men) on at least two occasions a month, being willing to attempt to reduce their alcohol consumption, owning a mobile phone and being willing to disclose their mobile phone number.

The invitation to the study will be sent by email including two reminders at 1 and 2 weeks after the initial invitation. Students will be allowed to respond up to 7 days after the last reminder. No other advertisement strategy will be used. The invitation email will aim to attract and reach students who think they drink too much and are willing to cut down. The invitation will be sent from the SHC.

Students can choose between two links in the email: (1) “Yes, I would like to know more about the study” or (2) “No, I do not wish to take part in the study or receive any reminders”. Students who click on the first link are referred to an eligibility criteria screen with one question: “How often during the last three months have you had more than four (male)/five (women) standard drinks of alcohol on one occasion?” There will be five response options: less than once a month/about once a month/2–3 times a month/about once a week/more than once a week.

Students who do not meet the inclusion criteria will be automatically referred to a screen including tips from a website where they can find support if needed. Students who meet the inclusion criteria will be automatically referred to an informed consent screen that also includes detailed information on the study and participation. Interested students will give their informed consent to participate by clicking on a link that will automatically refer them to a baseline questionnaire screen. The link contained within the body of the email is no longer valid after completion of the baseline questionnaire when the responses are stored in the study database. This prevents multiple responses but allows the questionnaire to be completed in more than one session if required.

**Fig. 2:**
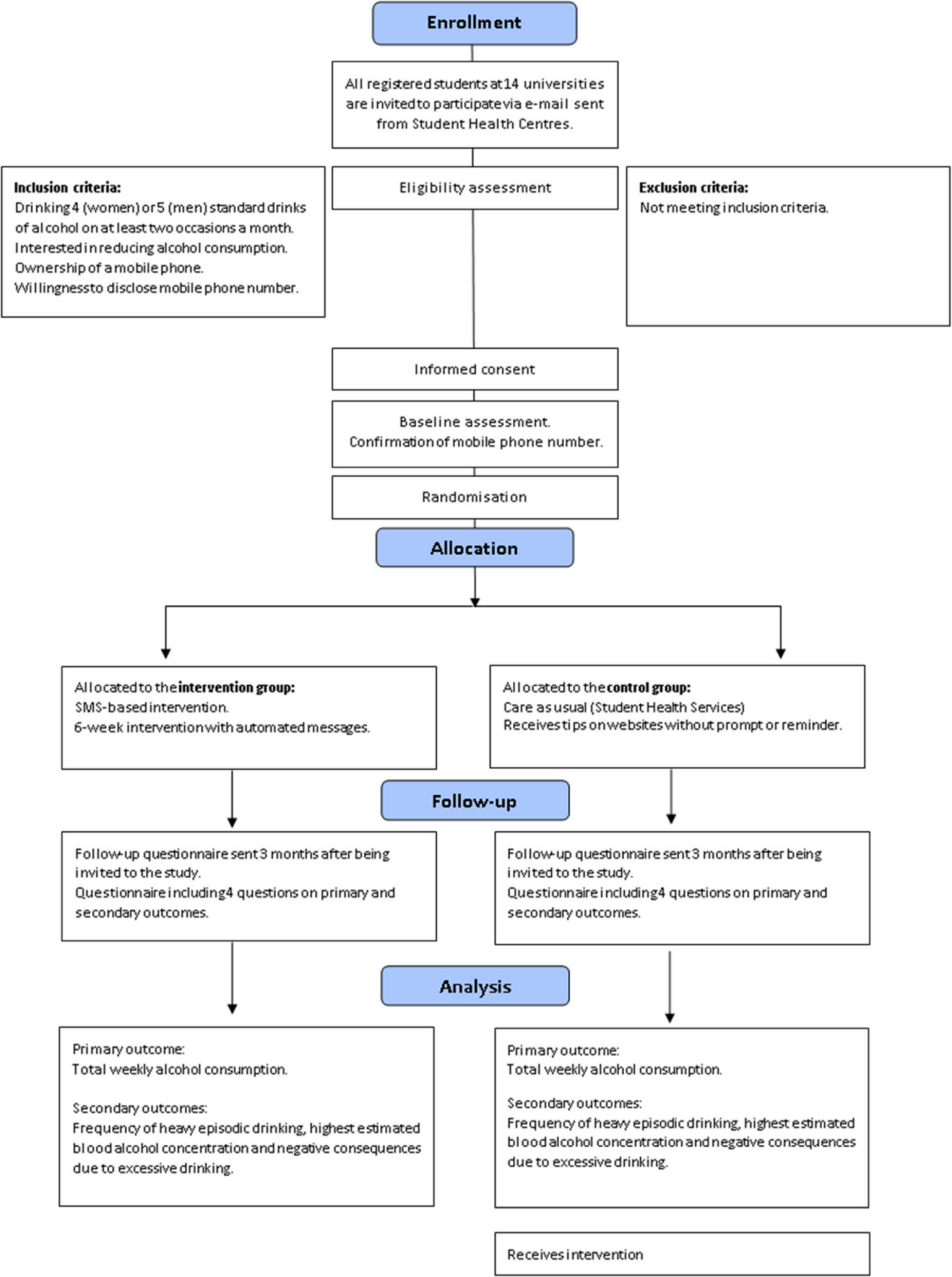
A flowchart of the recruitment procedure of the study

After completion of the baseline questionnaire, the students will be asked to provide their telephone number. Students will then immediately receive an SMS asking them to confirm their mobile phone number by responding “start”. All students who have confirmed their mobile phone number will be randomized to either the intervention or control group, SMS messages will be sent to each participant with information about which group they are allocated to. Figure 2 depicts a flowchart of the recruitment procedure of the study.

Follow up will be carried out 3 months after the initial invitation to the study. All participants will be sent an email invitation including a link to a follow-up questionnaire aiming to investigate the primary and secondary outcomes. Two reminders, 1 week apart, will be sent to non-responders, also by email. In addition, participants who still do not respond, will receive an SMS every second day for 6 days (that is three additional reminders) These SMS will only include a single question investigating the primary outcome (weekly consumption). Finally, those not responding to the SMS will be contacted by telephone (maximum of 10 calls). Again, only the primary outcome will be investigated.

### Baseline questionnaire

The baseline questionnaire will include a total of nine items. Items 1–3 will investigate (1) age (continuous); (2) sex (female/male) and (3) relationship status (single/in a relationship). Items 4 − 7 will investigate outcome measures for (4) total weekly alcohol consumption during a typical week; (5) HED during the last month; (6) eBAC during the last month and (7) number of negative consequences caused by drinking alcohol during the last month. In addition, students will be asked to (8) state their goal for reducing their weekly alcohol consumption and in combination with question 8, they will receive feedback on their weekly alcohol consumption, based on their earlier response. Finally, students are asked to (9) specify the mobile phone number to which they wish to receive the SMS support.

### Follow-up questionnaires

The follow-up questionnaire will include four questions investigating the primary and secondary outcomes: (1) total weekly alcohol consumption during a typical week; (2) HED during the last month; (3) eBAC during the last month and (4) number of negative consequences caused by drinking alcohol during the last month.

### Statistical analyses

The baseline characteristics of responders will be compared between randomized groups using the chi-squared test or Fisher’s exact test for comparison of proportions, and Student’s *t* test for comparison of means. All outcome analyses will be compared between the two randomized groups (both with the same follow-up time) under the intention-to-treat principle (that is, all randomized individuals will be included in their originally randomized groups).

Total weekly consumption, eBAC and the number of negative consequences will be assessed for skewness by visual inspection of histograms or Q-Q plots. If skewed, they will be either log-transformed and analysed with linear regression or analysed with negative binomial regression. Frequency of HED occasions will be analysed by ordered logistic regression. All regression analyses will first be performed unadjusted, and then will be adjusted for weekly alcohol consumption at baseline, age, university and gender. If any of the baseline characteristics are significantly different between the two groups, further adjustment will be made to these covariates as well. The adjusted analyses will be the primary analysis. The data will be examined graphically for outliers. A sensitivity analysis excluding outliers will be performed.

Missing outcome data will be initially handled by a complete-cases analysis, which assumes that the data are missing at random (MAR). If data are not MAR, then non-responders differ systematically from responders and early responders are likely to differ systematically from late responders, who are likely to be similar to non-responders. Therefore, we will explore the plausibility of the MAR assumption by first regression of the primary outcome (weekly alcohol consumption) on the number of follow-up attempts needed before an individual responded.

Second, attrition will be investigated for significant differences between the study groups in completion of the follow-up questionnaire, and baseline characteristics will be compared between participants who do and do not respond at follow up. Any significant associations found could provide possible evidence against the MAR assumption.

Effect modification tests for total weekly consumption according to baseline, age, university, and gender will be undertaken for the primary outcome only. All analyses will be performed with two-sided tests with a 5% level of significance.

### Power calculation

To detect an effect size of 15% standard deviations between the two groups at the 3-month follow up with a 5% significance level and 80% power, we require 699 individuals analysed per group, i.e. a total of 1398 individuals. Assuming a 3-month follow-up rate of 80%, we need 874 per group, i.e. a total of 1748 individuals.

## Discussion

Although heavy drinking of alcohol has significant health risks and presents negative social consequences, excessive drinking among university cultures is still a real problem (Thomas et al, in press). Student cultures with heavy drinking as the norm are prevalent both in Sweden and internationally. Considering the significant problem with heavy drinking in student populations, there is a need for the development and evaluation of accessible full-scale interventions that can reach a large proportion of the student population.

Previous studies have shown that a variety of automated Internet-based interventions have small, albeit significant, effects on a student’s drinking short term [16, 17, 19–22]. Thus, there is a need to further develop and evaluate new ways of reaching the large number of students who drink excessively.

Supporting evidence for the effect of SMS-based interventions is emerging; however, recent reviews have shown the need for RCTs that include larger sample sizes. This study will contribute with relevant knowledge on the effect of automated SMS support in student populations.

### Trial status

At the time of manuscript submission the recruitment of participants had not yet commenced; however, all plans and preparations for the trial were put in place.

## Additional files

Additional file 1: Complete list of final programme design SMS messages in Swedish and translated into English. (PDF 487 kb)

Additional file 2: SPIRIT checklist. (PDF 130 kb)

## Abbreviations

eBAC: Estimated blood alcohol concentration
HED: Heavy episodic drinking
RCT: Randomized controlled trial
SHC: Student Health Centre
SMS: Short message service
SPIRIT: standard protocol items: recommendation for interventional trials
