# Supplementary material for: Short message service (SMS)-based intervention targeting alcohol consumption among university students: study protocol of a randomized controlled trial
Source: Trials. 2017 Apr 4;18:156. doi: 10.1186/s13063-017-1898-3 (PMC5379716; doi:10.1186/s13063-017-1898-3)
Supplement: Supplementary file 1 — Complete list of final programme design SMS messages in Swedish and translated into English. (PDF 487 kb) [file 13063_2017_1898_MOESM1_ESM.pdf]

Additional file 1: Complete list of Final Programme Design SMS messages in Swedish and translated into English.

Message number	Timing Week, day, time	Swedish	English[1]	Behaviour change technique
1	1, 1, 3pm	Hej igen, hoppas du haft en bra helg! Nu börjar ditt deltagande i AMADEUS på riktigt.<br>För att kunna ge dig återkoppling ber vi dig beräkna hur många glas du har druckit den senaste veckan. Så här räknar du: ett s.k. standardglas motsvarar 1 flaska (33cl) starköl/cider/alkoläsk eller 1 litet glas vin (15cl), eller en liten shot sprit (4cl).<br>*Lägga en länk till bilder på standardglas.*<br>Svara på detta sms genom att endast ange en siffra, alltså antal standardglas du har druckit den senaste veckan.	Hi again<br>Hope you had a good weekend. You are now starting the AMADEUS programme.<br>In order to give you feedback on your progress we ask you to estimate how many standard drinks of alcohol you had the past week. One standard drink is: 1 bottle (33cl) of strong beer/cider or a small glass of wine (15cl) or a small shot of spirit (4 cl)<br>*Link to pictures of standard drinks*<br>In order to receive your feedback - respond to this SMS by writing the actual number of standard drinks only e.g. 8.	Provide feedback on performance
2	1, 1, 4pm	Bra att du har beräknat hur mycket du har druckit den senaste veckan. Hur ser det ut i förhållande till ditt mål som är xxx i veckan? Denna fråga kommer återkomma varje söndag för att du själv ska kunna följa din konsumtion.	Good that you keep track on your drinking. How does your current drinking correspond to the goal you set of a maximum of XXX standard drinks a week? This question is repeated each week in order for you to monitor your progress.	Provide feedback on performance
3	1, 2, 4pm	Du är nu på väg mot ditt mål. För att lättare nå ditt mål är det bättre att ta små steg åt gången än för stora kliv. Du kan t.ex. bestämma en gräns för hur mycket du dricker under en kväll.	You are now on your way towards reaching your goal. A tip is to take small steps, make small changes, towards your goal rather than embarking on big changes. For example, next time you’re out drinking, decide beforehand how many drinks you will have that evening. Make sure it’s less than you usually have and try to stick to your goal.	Provide instruction on how to perform the behaviour
4	1, 3, 4pm	Ett bra tips är att alltid äta innan eller samtidigt som du dricker alkohol. Om man dricker alkohol utan att ha ätit innan, kan det vara svårt att veta hur man regerar. Du kan bli mer berusad än du är van vid på samma mängd alkohol och därmed kanske utsätta dig för oönskade konsekvenser.	A good tip is to always eat some food before or during drinking. If you drink alcohol on an empty stomach, the alcohol can have a stronger, more negative, effect on you.	Provide information on consequences of excessive alcohol consumption and reducing excessive alcohol consumption
5	1, 3, 7pm	Om man ”tål alkohol bättre” än tidigare kan detta vara ett tecken på att man håller på att utveckla ett	If you tolerate alcohol better than previously did, it could be an indication that you are developing a dependence of alcohol.	Provide information on

		alkoholberoende då hjärnan kompenserar alkoholens dämpande effekt. Alkoholberoende kan som du vet innebära en hel del oönskade sociala, fysiska och psykiska konsekvenser.	This is because your brain always tries to compensate the inhibitory effects of alcohol. Alcohol dependence can, as you probably know, lead to a number of unwanted social, physical and psychological consequences.	consequences of excessive alcohol consumption and reducing excessive alcohol consumption
6	1, 4, 6pm	Varför dricker man alkohol? Det vanligaste är att man dricker för att bli glad och ha kul. Vissa dricker för att våga ta kontakt med andra medan andra dricker för att de man är tillsammans med dricker. Fundera på varför just du dricker alkohol? Gör du det alltid av egen fri vilja eller dras du med av kompistrycket?	Why drink alcohol? The most common reason is to relax and have fun. Some people drink to gain confidence whereas others 7drink only because their friends do. Take a moment to reflect w8hy you drink alcohol? Is it always your choice that you drink or is i9t sometimes the situations that prompt you to drink?	Identify reasons for wanting and not wanting to reduce excessive alcohol consumption
7	1, 4, 8pm	Om man dricker alkohol regelbundet i vissa situationer t.ex. efter en jobbig dag eller för att fira något kan det lätt bli en vana som innebär att man dricker alkohol utan att reflektera över det. Det bara blir så att man tar en öl eller liknande. Fundera på om det finns situationer där du nästan alltid dricker alkohol och att det skulle vara svårt att avstå?	If you always drink alcohol in particular situations for example after a difficult day, drinking can easily become a habit. You stop reflecting why you drink and it just becomes something you do. Take a moment now to reflect on which situations you most often drink alcohol and in which situations it would be difficult to say no to alcohol.	Assess current and past drinking behaviour
8	1, 5, 4pm	Man har alltid den bästa upplevelsen av alkohol precis när man börjar dricka. Detta beror på att dopamin är ett ämne i hjärnan som ger en känsla av upprymdhet och lycka. Dopamin frisätts under den första timman man dricker alkohol. Därefter frigörs inget mer dopamin oavsett hur mycket man dricker och upprymdhetskänslan försvinner.<br>Om du brukar dricka något glas innan du träffar dina kompisar – testa nästa gång att vänta tills ni träffas och känn efter hur den första positiva effekten av alkohol kommer när du är med dina vänner istället för ensam hemma. Ha en trevlig kväll!	You always experience the most positive effect of alcohol when having the first sip. This is due to a hormone in the brain, Dopamin, which gives you a feeling of exhilaration and happiness. Dopamin releases generally during den first hour you drink. Later on, no more Dopamin is released even if you continue to drink and the first effect of exhilaration and happiness disappears.<br>I you usually drink before meeting your friends – try to wait and drink. Look for the first positive effect of alcohol while you are with your friends instead of alone. Have a nice evening and stay safe.	Provide instruction on how to perform the behaviour
9	1, 6, 3pm	Ett tips som har fungerat för andra är att träna på att säga NEJ om du blir bjuden på alkohol. Tänk på ett scenario då du erbjuds alkohol och vad du kan säga som anledning till att tacka nej.	A tip that has worked for others is to practice to say NO if you are offered alcohol. Think on a situation where you could be offered alcohol and what reasons you could give for saying NO.	Prompt practice

10	1, 6, 6pm	Bra att du har bestämt dig för att minska din alkoholkonsumtion. Ett bra tips är att tala om för dina kompisar att du försöker minska din konsumtion. Det kan vara bra att ha vänner som känner till det och som du kan prata med.	It is good that you have decided to reduce your consumption. A good advice is to tell your friends that you are trying to reduce your drinking. It is good to have friends that are informed and that you can talk to, if you need to.	Advise on/facilitate use of social support
11	1, 7, 2pm	Risken att drabbas av en olycksfallsskada är 3-5 gånger större om man har 0,5 promille i blodet än om man är nykter. Om man dricker 3-4 öl på några timmars tid får man ungefär 0,5 promille i blodet. Det är dock stor individuell variation beroende på bl.a. faktorer som kön, vikt och vad man ätit innan.	The risk of having an accident is 3-5 times greater if you have a blood alcohol concentration of 0.5mg/ml compared with being sober. If you drink 3-4 small strong beers during a few hours you will have reached a blood alcohol concentration on at least 0.5mg/ml. However, the individual variation is large pending on sex, weight and what you have been eating before drinking alcohol.	Provide information on consequences of excessive alcohol consumption and reducing excessive alcohol consumption
12	2, 8, 2pm	Testade du att vänta med att börja dricka tills du träffade dina vänner i helgen? Upplevde du någon skillnad jämfört med tidigare?	Did you test to wait to drink until meeting with your friends this weekend? If so, did you experience a difference compared with your previous experience drinking alone?	Provide instruction on how to perform the behaviour
13	2, 8, 7pm	Då har det gått en vecka sedan du började AMADEUS. Förra söndagen fick du för första gången beräkna hur mycket du hade druckit under den gångna veckan. Nu är det dags igen. Vi räknar fortfarande i standardglas. Här kommer en påminnelse om hur man räknar. Ett standardglas motsvarar 1 flaska (33cl) starköl/cider/alkoläsk/1 litet glas vin (15cl), eller en liten shot (4 cl sprit).<br><br>*Länk till bilder på standardglas.*<br><br>Svara på detta sms genom att endast ange en siffra, alltså antal standardglas som du har druckit den senaste veckan.	It is now a week since you started the AMADEUS programme. Please estimate how many drinks you had the past week. We still calculate in standard drinks. Here is a reminder on how to calculate: One standard drink is: 1 bottle (33cl) of strong beer/cider or a small glass of wine (15cl) or a small shot of spirit (4 cl).<br>*Link to pictures of standard drinks*<br><br>In order to receive your feedback - respond to this SMS by writing the actual number of standard drinks only e.g. 8.	Provide feedback on performance
14	2, 8, 8pm	Bra att du har beräknat hur mycket du har druckit den senaste veckan. Hur ser det ut i förhållande till ditt mål som är xxx i veckan?	Good that you keep track on how much you drink. How does your drinking correspond with your goal of a maximum of XXX standard drinks a week?	Provide feedback on performance
15	2, 9, 4pm	Du visste säkert att det finns gott om kalorier i alkohol – dessutom kräver nedbrytningen av alkohol energi vilket gör att blodsockret sjunker och man blir onödigt hungrig.	You might already know that there are plenty of calories in alcohol – in addition drinking can lead to unnecessary hunger. As the body needs added energy to be break down the alcohol.	Provide information on consequences of

		Kan vara värt att tänka på om du vill hålla din vikt eller kanske gå ner i vikt.	This could be important to know if you want to keep your weight or lose weight. .	excessive alcohol consumption and reducing excessive alcohol consumption
16	2, 10, 5pm	När du har tid, ta några minuter och reflektera över vad som är viktigt i ditt liv och hur dina alkoholvanor kanske påverkar olika delar av livet som till exempel relationer, studier eller hälsa.	When you have time, take a few minutes to reflect on what is important in your life and how your alcohol habits affect these important aspects of your life for example relations, studies or your health.	Identify reasons for wanting and not wanting to reduce excessive alcohol consumption.
17	2, 11, 5pm	Om du någon gång har misslyckats med att minska din alkoholkonsumtion är du inte ensam då många har insett hur svårt det kan vara att ändra sina vanor. Samtidigt så är chansen större att lyckas nästa gång du prövar – så tveka inte att pröva igen.	If you have failed to reduce your drinking in the past you should know that you are not alone. Many before you know that it is difficult to change habits. On the other hand the chances that you will succeed next time you try is greater – so do not hesitate to try again.	Assess past history of attempts to reduce excessive alcohol consumption
18	2, 11, 7pm	Tidigare forskning säger att redan efter 4-5 flaskor starköl/små glas vin under en kväll utsätter man sig för en klar ökad risk att drabbas av en rad olika negativa konsekvenser såsom olycksfall, bråk, osams med vänner, sömnproblem oskyddat sex m.m. Kan något av detta stämma på dig?	Research has shown that after having consumed 4-5 bottles of strong beer or glasses of wine during an evening, you already increase the risk of negative consequences like accidents, fights, and arguments with friends, sleeping problems and unprotected sex, just to mention some of the risks. Do you recognise these risk?	Provide information on consequences of excessive alcohol consumption and reducing excessive alcohol consumption
19	2, 12, 12noon	Ett bra tips som har hjälpt andra är att inte bunkra upp för mycket alkohol hemma. Gör en lista på hur mycket du ska handla nästa gång du går till Systemet och handla inte mer än du skrivit ner.	A tip that others have benefitted from is to avoid stocking up alcohol at home. Make a list of what you need to buy the next time you go shopping and keep to the list.	Advise on environmental restructuring
20	2, 12, 6pm	Tips på ett litet experiment. I kväll eller nästa gång du ska dricka alkohol, testa att inte dricka till en början och säg till dina kompisar att du inte ska dricka denna gång. Observera hur dina kompisar reagerar på att du inte dricker.	Make a little experiment the next time you are going to drink with your friends. Tell them that you are not going to have any alcohol this time. Observe how you friends react when you abstain from drinking.	(Provide) information about other' approval
21	2, 13, 4pm	Utöver att man initialt blir upprymd när man börjar dricka alkohol så innebär ett fortsatt drickande att nästan alla delar av hjärnan påverkas på ett negativt eller dämpande sätt. Till exempel försämras hörseln även vid intag av mindre mängder – därför skruvas ljudnivån alltid upp i	Beside initial feelings of happiness when you drink, your brain will respond negatively to continuous drinking. All parts of the brain will be slowed down for example your hearing will be affected even after a small amount of alcohol – this is why a party drinking alcohol always is quite noisy.	Provide information on consequences of excessive alcohol consumption and

		sällskap där det dricks alkohol. Kolla själv nästa gång du är med i ett sällskap som dricker alkohol.	You could try to notice this the next time you drink with your friends.	reducing excessive alcohol consumption
22	2, 14, 3pm	Har någon av dina kompisar kommenterat dina alkoholvanor genom t.ex. att påpeka att du dricker för mycket eller för ofta? Vad tycker du själv och finns det någon relevans i dina kamraters påstående – kan du göra något åt detta?	Have any of your friends commented on that you drink too much or too often? Do you agree with them? Is there anything you could do about this?	(Provide) information about other' approval
23	3, 14, 2pm	Genomförde du experimentet i helgen? Om inte, kan du alltid göra det nästa gång du ska dricka alkohol i sällskap med dina vänner. Kan bli en intressant upplevelse. Ha en trevlig eftermiddag.	Did you try to stay off the alcohol when meeting with your friends this weekend? If not you could perhaps do this the next time you drink with them. It could be an interesting experience. Have a nice afternoon.	(Provide) information about other' approval
24	3, 15, 7pm	Go' kväll. Nu har det gått två veckor sedan du började AMADEUS.<br>För att kunna ge dig återkoppling på hur det går ber vi dig beräkna hur många glas du har druckit den senaste veckan. Så här räknar du, ett s.k. standardglas motsvarar: 1 flaska (33cl) starköl/cider/alkoläsk eller ett litet glas vin (ca.15cl) eller en liten shot sprit (4cl).<br>*En länk till bilder på standardglas.*<br>Svara på detta sms genom att endast ange en siffra, alltså antal standardglas du har druckit den senaste veckan.	Good evening. It is now two weeks since you started the AMADEUS programme.<br>Please estimate the number of drinks you had the past week. We still calculate in standard drinks. Here is a reminder on how to calculate: One standard drink is: 1 bottle (33cl) of strong bear/cider or a small glass of wine (15cl) or a small shot of spirit (4 cl)<br>*Link to pictures of standard drinks*<br>In order to receive your feedback - respond to this SMS by writing the actual number of standard drinks only e.g. 8.	Provide feedback on performance
25	3, 15, 8pm	Bra att du har koll på hur mycket du har druckit den senaste veckan. Hur ser det ut i förhållande till ditt mål som är xxx i veckan?	Good that you have calculated how many drinks you had the past week. How does your current drinking correspond to the goal you set of a maximum of XXX standard drinks a week?	Provide feedback on performance
26	3, 16, 4pm	Samma mängd alkohol påverkar personer olika. Exempelvis så får en stor person mindre promillehalt i blodet jämfört med en person som är mindre eller väger mindre. Kvinnor får 20-30% högre promille vid samma intag av alkohol som en man pga. att de oftast väger mindre och har mindre andel vatten i kroppen jämfört med män.	Similar amount of alcohol influence persons differently. For example a bigger person will get a lower blood alcohol 27concentration compared with a smaller person. Also, women no28rmally will reach a 20-30 % higher blood alcohol conc29entration compared to a man even if they drink the same amoun30t of alcohol. This is due to that women often weight less and h31ave a smaller proportion of water in their body composition32 compared with men.	Provide information on consequences of excessive alcohol consumption and reducing excessive alcohol consumption
27	3, 17, 4pm	Om du känner dig pressad och stressad och får lust att " bara dricka bort allt", försök med alternativa sätt att få	If you often fee33l pressured or stressed and get a desire to "drink and forget"34, try alternative means of letting go for	Behaviour substitution

		bort stressen t.ex. genom en frisk promenad eller en bra film. Gör helt enkelt något som får dig att må bra!	example take a good35 long walk or watch a movie. Try to do things that you know m36akes you feel better.	
28	3, 18, 3pm	Ett tips som fungerat för andra: innan du träffar dina kompisar nästa gång, tänk ut vad du ska säga om de frågar varför du inte dricker som du brukar.	A tip that have worked for others: before you meet with your friends next time prepare an answer to why you drink less alcohol than you normally do.	Prompt practice
29	3, 18, 7pm	Alkohol påverkar sömnen negativt. Känner du igen dig i att man kanske inte kan somna alls eller somnar snabbt när man har druckit mycket men vaknar ofta redan efter några timmar utan att kunna somna om? Det beror på att kroppen arbetar för att alkoholen ska lämna blodet och när det börjar ske försvinner den sövande effekten och man vaknar. Om man ofta dricker en större mängd alkohol kan det leda till störd sömn och kronisk trötthet.	Alcohol affects sleep negatively. Do you recognise that it is difficult to fall asleep after drinking or that you fall asleep quickly but wake up after a few hours and are not able to get back to sleep? This is because your body works hard to get rid of the alcohol and when alcohol is broken down the sedating effects disappear. If you often drink a lot of alcohol this could lead to disturbed sleep and chronic fatigue.	Provide information on consequences of excessive alcohol consumption and reducing excessive alcohol consumption
30	3, 19, 8pm	Ett bra sätt att undvika tillfällen där du dricker mer än du hade tänkt dig är att fundera på vad som händer vid dessa tillfällen och vad du kan göra för att undvika detta. Fundera på en situation nyligen då du drack på ett sådant sätt som inte blev så bra. Formulera en mening för dig själv som börjar med ”om jag hade druckit mindre så hade…”. Hur kan du undvika liknande situationer i framtiden tror du?	A good way to avoid occasions when you drink more than you want to, is to reflect what is happening on these occasions and try to identify what you can do to change things. Think of a recent occasion when you drank to excess. Try to formulate a sentence “if I had been drinking less, then……” Also, how can you avoid similar situation in the future?	Identify reasons for wanting and not wanting to reduce excessive alcohol consumption
31	3, 20, 4pm	Om du känner dig orolig, nedstämd eller helt enkelt inte mår så bra kommer du troligtvis må ännu sämre om du dricker alkohol. Alkohol förstärker ofta den sinnesstämning man är i när man börjar dricka.	If you feel anxious, depressed or low in general you will probably feel even worse after drinking alcohol. This is because alcohol enhances your current mood.	Provide information on consequences of excessive alcohol consumption and reducing excessive alcohol consumption
32	3, 21, 3pm	Bra att du kämpar på med en minskning av din alkoholkonsumtion. Finns det något som försvårar en ändring av dina alkoholvanor? Vad kan du göra åt detta? Finns det något som du vet har fungerat för dig tidigare?	Good that you keep on trying to reduce your drinking. Is there anything that complicates your efforts to reduce your drinking? What can you do about this? Is there anything that have worked previously that also could try now?	Facilitate barrier identification and problem-solving
33	3, 21, 5pm	I kväll eller nästa gång du skall dricka alkohol bestäm dig för att dricka ”varannan vatten”, då kommer du att må	Tonight or next time you’re drinking decide to drink “every second water”, then you will feel much better the next day. Guaranteed. And you will save money. Have a nice evening.	Provide instruction on how to perform the behaviour

		bättre dagen därpå. Garanterat. Och du sparar en del pengar. Ha en trevlig kväll.		
34	4, 22, 7pm	Go kväll. Hoppas att du har haft en fin helg. Om du var ute på krogen och drack alkohol i helgen – ta dig en funderare på hur mycket pengar det har kostat dig? Var även de sista drinkarna värda pengarna eller hade du redan fått tillräckligt?<br>Vi ber dig som vanligt varje söndagskväll sms:a hur många standardglas som du har druckit den senaste veckan. Redo? 1 standardglas motsvarar 1 flaska (33cl) starköl/cider/alkoläsk/ett litet glas vin (15cl), eller en liten shot sprit (4cl).<br>*Länk till bilder på standardglas*<br>Svara på detta sms genom att endast ange en siffra, alltså antal standardglas som du har druckit den senaste veckan.	Good evening. Hope that you had a good weekend. If you were out drinking this weekend – take a little time and think how much money you spent on alcohol?<br>Now it is time again to calculate how many drinks you had last week. We still calculate in standard drinks. Here is a reminder on how to calculate: One standard drink is: 1 bottle (33cl) of strong beer/cider or a small glass of wine (15cl) or a small shot of spirit (4 cl).<br>*Link to pictures of standard drinks*<br>In order to receive your feedback - respond to this SMS by writing the actual number of standard drinks only e.g. 8.	Provide feedback on performance
35	4, 22, 8pm	Bra att du har koll på hur mycket du har druckit den senaste veckan. Hur ser det ut i förhållande till ditt mål som är xxx i veckan?	Good that you keep track on your drinking. How does your current drinking match the goal you set at the start of AMADEUS of a maximum of XXX standard drinks a week?	Provide feedback on performance
36	4, 23, 5pm	Nu har du kommit halvvägs i programmet. Reflektera över hur nöjd du är med dina alkoholvanor på en skala från 1 (inte nöjd alls) till 10 (mycket nöjd).	Now you are half way in the AMADEUS programme. Take a little time to reflect how satisfied you are with your current alcohol habits – use a scale from 1 (not satisfied at all) to 10 (very satisfied).	Prompt review of goals
37	4, 23, 7pm	Om du är missnöjd med dina vanor i testet som vi beskrev i det senaste sms:et – vad drar du för slutsatser? Om du är nöjd med dina alkoholvanor – vad kan det beror det på?	If you are dissatisfied with your alcohol habits – what could you do about it? If you are satisfied with your habits – why is it so?	Prompt review of goals
38	4, 24, 6pm	Om du känner att du har ”förtjänat” ett par öl/drinkar efter en ansträngande vecka, försök komma på andra sätt att belöna dig själv; det kan vara en lyxig tidning, god mat eller vad du känner är något lite extra ”utöver det vanliga”.	If you feel that you have “earned” a couple of beers or drinks after a busy week, try to find other means of rewarding yourself. Buy an interesting magazine, good food or anything that you think is a treat.	Behaviour substitution
39	4, 25, 7pm	Vissa dricker alkohol för att slappna av och minska stressen från de krav de upplever från omgivningen eller som de själva satt upp. Det är dock riskfyllt att börja använda alkohol för att minska stress. Bättre vore förstås att fundera på hur du kan minska den stress du upplever i	Some people drink alcohol as a stress relief. It is a risky business to use alcohol as a coping strategy. It is of course better to use other means of relaxing. Using alcohol often leads to more problems and more stress.	Provide information on consequences of excessive alcohol consumption and

		ditt liv. Att använda alkohol leder i många fall bara till mer problem och stress.		reducing excessive alcohol consumption
40	4, 26, 2pm	Tips på reflektion: om du fortfarande dricker mer alkohol än du har tänkt dig, fundera i några minuter på hur du skall undvika detta i fortsättningen och pröva att följa din plan nästa gång du dricker.	Tip: if you still are drinking more alcohol than you wished, take some time once again and reflect how you can avoid this in the future. Make a new effort to follow your intention the next time you drink.	Facilitate barrier identification and problem-solving
41	4, 26, 5pm	Om du brukar förfesta bestäm dig nästa gång för hur mycket du skall dricka och ta inte med mer alkohol än vad du tänkt dig. Fundera också på hur länge förfesten troligtvis håller på så att du kan planera att dricka i lugn takt under festen.	If you usually start drinking with friends early, or before an evening out, next time try to decide beforehand how much you are drinking and only bring this amount. Also try to think how long you will be out so you can plan to drink slowly.	Provide instruction on how to perform the behaviour
42	4, 27, 4pm	I kväll eller nästa gång du ska dricka alkohol försök att dricka långsammare än du brukar. Bestäm dig för hur lång tid det skall ta att dricka t.ex. en öl eller glas vin. Då kommer du garanterat att vara piggare hela kvällen och får en mindre mängd alkohol i kroppen. Ha en trevlig kväll.	Tonight or next time you drink, try to drink a bit slower than usual. Decide beforehand how long it will take to drink a beer or a glass of wine. You will be more alert throughout the evening and you will end up drinking less. Have a nice evening and take care of yourself.	Provide instruction on how to perform the behaviour
43	4, 27, 9pm	Om du är ute och festar ikväll och dricker alkohol kom i så fall ihåg att dricka ”varannan vatten”! Du kommer må bättre imorgon och sparar troligtvis en del pengar. Ha en fortsatt trevlig kväll.	If you are out drinking tonight don´t forget to “drink every second water”. You will feel better tomorrow and you will probably save some money. Have a nice evening and stay safe.	Provide instruction on how to perform the behaviour
44	4, 28, 4pm	Forskningen säger att om man tränar hårt och vill ha bra resultat bör man undvika ett större intag av alkohol en till två dagar innan man tränar, då alkoholen inverkar negativt på musklernas energiförsörjning som är viktig för muskeluppbyggandet.	Research have shown that if you exercising extensively and want good results you should avoid drinking too much one to two days before your workout. This is due to that alcohol influences negatively on the energy supply to the muscles which is important for building muscles.	Provide information on consequences of excessive alcohol consumption and reducing excessive alcohol consumption
45	5, 29, 7pm	Vi ber dig som vanligt varje söndagskväll sms:a hur många standardglas som du har druckit den senaste veckan. Redo? 1 standardglas motsvarar 1 flaska (33cl) starköl/cider/alkoläsk eller ett litet glas vin (15cl), eller en liten shot sprit (4cl). *Länk till bilder på standardglas*	Now it is time again to calculate how many drinks you had last week. We still calculate in standard drinks. Here is a reminder on how to calculate: One standard drink is: 1 bottle (33cl) of strong bear/cider or a small glass of wine (15cl) or a small shot of spirit (4 cl). *Link to pictures of standard drinks*	Provide feedback on performance

		Svara på detta sms genom att endast ange en siffra, alltså antal standardglas som du har druckit den senaste veckan.	In order to receive your feedback - respond to this SMS by writing the actual number of standard drinks only e.g. 8.	
46	5, 29, 8pm	Bra att du har koll på hur mycket du har druckit den senaste veckan. Hur ser det ut i förhållande till ditt mål som är xxx i veckan?	Good that you keep track on how much you drink. How does your current drinking correspond to the goal you set of a maximum of XXX standard drinks a week?	Provide feedback on performance
47	5, 30, 10am	Go'morron. Var du ute på krogen och drack alkohol i helgen? Hur mycket pengar uppskattar du att du lägger på alkohol på en vecka. Fundera på hur mycket pengar du spenderar på alkohol jämfört med mat under en vecka.	Good morning. Were you out drinking this weekend? How much do you spend on alcohol each week? Take a moment to think how much money you spend on alcohol compare to food and groceries a typical week.	Assess current and past drinking behaviour
48	5, 30, 6pm	Det har nu gått 4 veckor sedan du började AMADEUS. Om man umgås med vänner som dricker mycket och ofta kan det vara svårt att minska sin egen alkoholkonsumtion. Det kan underlätta att undvika vissa vänner på festkvällar och umgås dagtid istället.	It is now 4 weeks since you started the AMADEUS programme. If you hang out with friends who drink a lot, it could be more difficult to reduce your own drinking. It could be helpful to avoid certain friends on nights out.	Advise on avoidance of social cues for drinking
49	5, 31, 5pm	Om man ofta funderar på när man ska dricka alkohol nästa gång kan detta vara ett tecken att man håller på att utveckla ett beroende som kan vara svårt att bryta. Alkoholberoende är ett tillstånd som medför stora negativa sociala, fysiska och psykiska konsekvenser.	If you often think about when it's time to drink again, this could be a warning signal that you are developing a dependence of alcohol. Alcohol dependence leads to serious negative social, physical and mental consequences.	Provide information on consequences of excessive alcohol consumption and reducing excessive alcohol consumption

50	5, 32, 7pm	Olika personer har olika förväntningar på vad som händer när man dricker alkohol. Dessa förväntningar kan vara en drivkraft att dricka eller en anledning till att man avstår att dricka.<br><br>Hur bra stämmer dessa påståenden på dig?<br><br>När jag dricker alkohol blir jag mer oförsiktig.<br>När jag dricker bryr jag mig mindre om konsekvenserna av mina handlingar.<br>När jag dricker blir jag lättare irriterad på min omgivning.<br>När jag dricker får jag svårt att koncentrera mig.<br>När jag dricker alkohol hamnar jag lättare i bråk.<br><br>Om du kommer fram till att alkohol har många negativa konsekvenser bör du fundera på att ibland undvika att dricka eller begränsa din konsumtion.	Different persons have different expectations to what is happening when they drink alcohol. These expectations could motivate to drink or motivate to stay off the alcohol.<br><br>How do these statements correspond to your situation?<br><br>When I drink I get more careless.<br>When I drink I care less about the consequences<br>When I drink I get easily irritated<br>When I drink I have difficulty to concentrate<br>When I drink I easier get into an argument<br><br>If you realize that alcohol gives you many negative consequences you ought to consider to reduce or stop drinking.	Identify reasons for wanting and not wanting to reduce excessive alcohol consumption
51	5, 33, 4pm	Om du just nu eller längre fram har en period där du dricker mer alkohol än du egentligen hade tänkt dig så var inte rädd att söka hjälp och stöd från din omgivning som kompisar, familj eller professionell hjälp. Det är många fler än du som har behövt söka hjälp.<br><br>Mer hjälp finns att få på www.alkoholhjalpen.se eller på din Vårdcentral.	If you currently or later in life go through a phase of drinking more than you wish to, do not hesitate to seek support from family, friends or professional help. Many more before you have had to seek help.<br>More help can be found on www.alkoholhjalpen.se or you can ask your doctor for guidance and more information.	5, 33, 4pm
52	5, 33, 6pm	I kväll eller nästa gång du ska dricka alkohol bestäm dig innan för hur mycket du skall dricka totalt. Försök sen att under kvällen hålla räkning på hur mycket du dricker. Ha en trevlig kväll! Kom ihåg att dricka ”varannan vatten”.	Tonight or the next time you are going to drink alcohol, decide beforehand how much you are going to drink. Try to keep track of your drinking during the evening. Have a nice evening. Remember to have every second water.	Prompt self-recording
53	5, 35, 4pm	Om du någon gång har tänkt så här: " Minskar jag snart inte mitt drickande kommer jag att få problem" bör du kanske överväga att börja ändra dina alkoholvanor - eller hur?	Have you ever had a thought like this: “If I don’t reduce my drinking soon I will develop a problem?” If so, then you ought to consider reducing your drinking – wouldn’t you agree?	Identify reasons for wanting and not wanting to reduce excessive alcohol consumption

54	6, 36, 2pm	Försökte du hålla räkning på hur mycket du drack i helgen? Hur gick det, höll du dig till ditt eget mål? Om inte, vad drar du för slutsats?	Did you try to keep track of how much you were drinking this weekend? How did it go? Did you manage to stay within your limits? If not – what is your thoughts on this?	Prompt self-recording
55	6, 36, 7pm	Go kväll. Hoppas att du har haft en fin helg. När du får tid, kan du kanske ha nytta av att reflektera över om ditt drickande ibland medför att du inte uppnår vissa mål du har satt i din vardag?<br>Vi ber dig som vanligt varje söndagskväll sms:a hur många standardglas som du har druckit den senaste veckan.<br>Svara på detta sms genom att endast ange en siffra, alltså antal standardglas som du har druckit den senaste veckan.<br>*Länk till bilder på standardglas*<br>Redo? 1 standardglas motsvarar 1 flaska (33cl) starköl/cider/alkoläsk/ ett litet glas vin (ca.15cl), eller en liten shot sprit (4cl).<br>Svara på detta sms genom att endast ange en siffra, alltså antal standardglas som du har druckit den senaste veckan.	Good evening. Hope that you have had a nice weekend. When you find time it is a good idea to consider whether your drinking 56habits mean that you are sometimes not able to fulfil your own personal goals.<br>Now it is time again to calculate your past week drinking. We still calculate in standard drinks. Here is a reminder on how to calculate: One standard drink is: 1 bottle (33cl) of strong bear/cider or a small glass of wine (15cl) or a small shot of spirit (4 cl).<br>*Link to pictures of standard drinks*<br>In order to receive your feedback - respond to this SMS by writing the actual number of standard drinks only e.g. 8.	Provide feedback on performance
56	6, 36, 8pm	Bra att du har koll på hur mycket du har druckit den senaste veckan. Hur ser det ut i förhållande till ditt mål som är xxx i veckan?	Good that you keep track on how much you drink. How does your current drinking correspond to the goal you set of a maximum of XXX standard drinks a week?	Provide feedback on performance
57	6, 37, 6pm	Du är inne på din sjätte och sista vecka i AMADEUS. Har du tänkt på varför du dricker den mängd alkohol som du gör? Är det för att du vill det eller för att du känner dig tvungen i sällskap med dina kompisar?	You are now on the 6th and last week of the AMADEUS programme. Have you had time to consider why you drink the amount of alcohol you do? Is it because you have chosen the amount or is it because you feel pressured when meeting with your friends?	Emphasize choice
58	6, 39, 6pm	Om du just nu eller längre fram har/får en period där du dricker mer alkohol än du egentligen hade tänkt dig: bestäm dig för att inte bunkra upp med mycket alkohol hemma.	If you right now or later in life have a period when you drink more than you want to, make sure not to pile up too much alcohol at home.	Advise on environmental restructuring
59	6, 41, 4pm	I kväll eller nästa gång du ska dricka alkohol försök att dricka svagare drinkar eller byt till dryckessorter med mindre alkohol och/eller mindre storlek och känn efter hur du mår nästa dag. Ha en trevlig kväll.	Tonight or next time you are drinking try to choose drinks with less alcohol. Take time to reflect on how you feel the next day. Have a nice evening.	Prompt practice

60	6, 42, 4pm	Go kväll. Hoppas att du har haft en fin helg. När du har tid – nu eller kanske senare, reflektera över hur det vanligtvis känns dagen efter du har druckit en större mängd alkohol? Finns det något som du är missnöjd med och skulle vilja förändra?	Good evening. Hope that you have had a nice weekend. When you got time – now or later – reflect on how you usually feel the day after drinking a large amount of alcohol. Is there anything you wished were different or that you want to change?	Anticipated regret
61	7, 43, 7pm	Nu har det gått 6 veckor sedan du började AMADEUS.<br>Vi hoppas att du har lärt dig mer om varför du dricker och hur du på olika sätt kan minska ditt drickande. Vi ber dig en sista gång sms:a hur många standardglas som du har druckit den senaste veckan. Svara på detta sms genom att endast ange en siffra, alltså antal standardglas som du har druckit den senaste veckan.<br>Redo? 1 standardglas motsvarar 1 flaska (33cl) starköl/cider/alkoläsk eller ett litet glas vin (ca.15cl), eller en liten shot sprit (4cl).<br><br>*Länk till bilder på standardglas*<br>Svara på detta sms genom att endast ange en siffra, alltså antal standardglas som du har druckit den senaste veckan.	It is now 6 weeks since you started the AMADEUS programme. We hope that you have learnt more about your drinking habits and how you can reduce your alcohol consumption.<br><br>Now it is the last time when we ask you to calculate your past week drinking. We still calculate in standard drinks. Here is a reminder on how to calculate: One standard drink is: 1 bottle 62(33cl) of strong bear/cider or a small glass of wine (15cl) or a sm63all shot of spirit (4 cl).<br>*Link to pictures of standard drinks*<br>In order to receive your feedback - respond to this SMS by writing the actual number of standard drinks only e.g. 8.	Provide feedback on performance
62	7, 43, 8pm	Har du nu efter 6 veckor nått ditt mål? Om inte är det bara att fortsätta kämpa. Kan ta tid att få till nya vanor. Tack för ditt deltagande i Amadeus sms-stöd-programmet. Härmed avslutas programmet men om du vill ha mer hjälp hänvisar vi till www.alkoholhjalpen.se	Have you after these 6 weeks reached your goal in reducing your drinking? If not we urge you to keep on trying. It can take time to establish new habits.<br>Thank you for participating in the AMADEUS programme. This is the last SMS but if you feel you would like more help and support check out www.alkoholhjalpen.se.	Provide feedback on performance

[1]English translation for the purpose of this paper only.
